# Supplementary material for: The Relationship between Neuropsychiatric Symptoms and Cognitive Performance in Older Adults with Normal Cognition
Source: Medicina (Kaunas). 2022 Nov 3;58(11):1586. doi: 10.3390/medicina58111586 (PMC9694960; doi:10.3390/medicina58111586)
Supplement: Supplementary file 1 [file medicina-58-01586-s001.zip › medicina-1899791-supplementary.pdf]

Table S1 Associations between neuropsychiatric manifestations and cognitive performance: confirmatory analysis.

NPS		Episodic memory		Language		Semantic Memory		Attention		Processing speed		Executive function	
		B	p	B	p	B	p	B	p	B	p	B	p
Depression	0-1	.291	.690	.528	.493	-.302	.307	.094	.628	1.761	.224	1.872	.675
	2-3	Ref		Ref		Ref		Ref		Ref		Ref	
Anxiety	0-1	1.227	.090	-.739	.334	**.883(1)**	**.003**	**.377(2)**	**.049**	-1.726	.230	**-10.424(3)**	**.019**
	2-3	Ref		Ref		Ref		Ref		Ref		Ref	
Agitation	0-1	.652	.451	.024	.979	-.280	.425	.021	.926	-1.209	.481	-2.081	.694
	2-3	Ref		Ref		Ref		Ref		Ref		Ref	
Apathy	0-1	.872	.459	1.563	.209	-.021	.966	.161	.606	-.915	.695	-2.636	.714
	2-3	Ref		Ref		Ref		Ref		Ref		Ref	
Disinhibition	0-1	.358	.772	-.191	.884	.046	.928	.030	.927	1.017	.678	-9.671	.200
	2-3	Ref		Ref		Ref		Ref		Ref		Ref	
Irritability	0-1	.762	.268	-.167	.818	-.057	.837	-.176	.333	-2.408	.077	-2.558	.543
	2-3	Ref		Ref		Ref		Ref		Ref		Ref	
Night-time	0-1	.472	.411	.716	.237	.143	.540	.071	.642	.849	.456	4.179	.233
	2-3	Ref		Ref		Ref		Ref		Ref		Ref	
Appetite	0-1	.268	.744	1.412	.103	.589	.077	.144	.508	-2.530	.120	-8.696	.083
	2-3	Ref		Ref		Ref		Ref		Ref		Ref	

Elation	No	1.148	.296	.917	.429	.197	.659	**.740[4]**	**.011**	-2.421	.267	-4.819	.473
	Yes	Ref		Ref		Ref		Ref		Ref		Ref	
Motor	No	1.662	.073	**2.420[5]**	**.013**	-.222	.554	.094	.702	1.719	.350	-.870	.878
	Yes	Ref		Ref		Ref		Ref		Ref		Ref	
Delusions	No	1.025	.400	.966	.453	.457	.355	.431	.181	-2.265	.349	-3.933	.597
	Yes	Ref		Ref		Ref		Ref		Ref		Ref	
Hallucinations	No	3.825	.082	.084	.971	.404	.651	.097	.868	**-10.702[6]**	**.014**	**-45.112[7]**	**.001**
	Yes	Ref		Ref		Ref		Ref		Ref		Ref	

Ref: reference group; the numbering of the neuropsychiatric symptoms corresponds to: 0-1= absent-mild; and 2-3 moderate-severe symptomatology; 95%CIs [1] (.307, 1.458); [2] (.001, .753); [3] (-19.101, -1.747); [4] (.169, 1.310); [5] (.502, 4.338); [6] (-19.255, -2.148); [7] (-71.473, -18.750)

Table S2 Associations between neuropsychiatric manifestations and cognitive performance: exploratory analysis.

NPS		Episodic memory		Language		Semantic Memory		Attention		Processing speed		Executive function	
		B	p	B	p	B	p	B	p	B	p	B	p
Depression	0-1	.218	.765	.423	.582	-.326	.269	.116	.549	2.148	.137	3.324	.456
	2-3	Ref		Ref		Ref		Ref		Ref		Ref	
Anxiety	0-1	1.269	.080	-.583	.446	**.914(1)**	**.002**	**.385(2)**	**.045**	-1.876	.192	**-11.172(3)**	**.012**
	2-3	Ref		Ref		Ref		Ref		Ref		Ref	
Agitation	0-1	.771	.370	.231	.799	-.210	.547	.046	.842	-1.180	.489	-3.128	.552
	2-3	Ref		Ref		Ref		Ref		Ref		Ref	
Apathy	0-1	.760	.520	1.519	.223	-.013	.978	.162	.604	-1.040	.657	-3.456	.632
	2-3	Ref		Ref		Ref		Ref		Ref		Ref	
Disinhibition	0-1	.383	.758	-.170	.897	.170	.735	.041	.900	1.513	.538	-9.586	.207
	2-3	Ref		Ref		Ref		Ref		Ref		Ref	
Irritability	0-1	.689	.319	-.204	.780	-.085	.762	-.170	.352	-2.386	.081	-2.373	.574
	2-3	Ref		Ref		Ref		Ref		Ref		Ref	
Night-time	0-1	.503	.382	.850	.161	.163	.483	.067	.659	.640	.574	3.406	.332
	2-3	Ref		Ref		Ref		Ref		Ref		Ref	
Appetite	0-1	.209	.799	1.366	.116	.620	.063	.147	.500	-1.788	.273	-7.775	.123
	2-3	Ref		Ref		Ref		Ref		Ref		Ref	

Elation	0-1	1.990	.336	2.514	.250	-.735	.381	**1.228**[4]	**.025**	**-12.604**[5]	**.002**	-6.308	.618
	2-3	Ref		Ref		Ref		Ref		Ref		Ref	
Motor	0-1	2.946	.129	.832	.685	-.093	.906	.291	.571	1.830	.634	-5.517	.642
	2-3	Ref		Ref		Ref		Ref		Ref		Ref	
Delusions	0-1	4.098	.100	3.859	.142	1.428	.157	.236	.720	**-14.262**[6]	**.004**	**-33.401**[7]	**.028**
	2-3	Ref		Ref		Ref		Ref		Ref		Ref	
Hallucinations	0-1	5.923	.265	-3.027	.589	-.630	.770	.808	.566	16.674	.113	16.745	.606
	2-3	Ref		Ref		Ref		Ref		Ref		Ref	

Ref: reference group; the numbering of the neuropsychiatric symptoms corresponds to: 0-1 absent-mild; and 2-3= moderate-severe symptomatology; 95%CIs [1] (.338, 1.490); [2] (.008, .761); [3] (-19.862, -2.481); [4] (.154, 2.302); [5] (-20.642, 4.566); [6] (-23.925, -4.598); [7] (-63.216, -3.585)
